# Understanding Attrition and Bolstering Retention in a Longitudinal Panel of Older Adults: ORANJ BOWL

**DOI:** 10.1093/geroni/igab010

**Published:** 2021-03-23

**Authors:** Allison R Heid, Francine P Cartwright, Maureen Wilson-Genderson, Rachel Pruchno

**Affiliations:** 1 Ardmore, Pennsylvania, USA; 2 New Jersey Institute for Successful Aging, Rowan University School of Osteopathic Medicine, Stratford, New Jersey, USA; 3 Short Hills, New Jersey, USA

**Keywords:** Biased attrition, Developmental research, Quantitative research methods, Research methods, Successful aging

## Abstract

**Background and Objectives:**

Attrition from longitudinal studies can affect the generalizability of findings especially when studying developmental constructs such as successful aging.

**Research Design and Methods:**

Using data from a 12-year (6-wave) panel of 5,688 older people (aged 50–74 at baseline), we compared people retained in the panel with people lost to follow-up on demographic characteristics and measures of successful aging. After instituting expanded retention strategies at Wave 6 (i.e., a team-based approach, social media, and paid web search engines), we compared different groups of people lost to follow-up (i.e., deceased and withdrawn due to lack of interest) and different types of completers (i.e., full completers vs. lost and reengaged completers).

**Results:**

At baseline, Wave 6 completers were significantly younger, less likely to be African American, more likely to be married, reported higher levels of income and education, were more likely to be working full-time, had less pain and fewer chronic illnesses, and reported higher levels of subjective successful aging and functional ability than those lost to follow-up. Analyses demonstrated differences across groups based on the reason for loss (i.e., deceased, impaired, and not interested). Participants who missed an interview but returned to the panel were significantly different from those who participated in all waves of data collection. Expanded retention efforts improved generalizability, as people returning to the panel reported lower levels of education, lower levels of income, and were more likely to be African American.

**Discussion and Implications:**

Biased attrition within longitudinal research affects the interpretation of study findings, especially when studying developmental outcomes. However, expanded retention strategies can reduce bias and loss and should be used to enhance retention efforts in longitudinal work.

**Translational Significance:** These findings inform research scientists about the benefits of using creative, flexible retention strategies in longitudinal aging research in order to maintain as much sample diversity as possible and reduce bias due to selective attrition. The use of standard retention efforts (i.e., regular mailings) combined with expanded retention efforts (i.e., a team-based approach to data collection, access to social media, and use of paid deep-web search engines) can reduce biased attrition over time.

## Background and Objectives

Retaining samples of people in longitudinal aging research that is unbiased because of attrition patterns is key to understanding developmental outcomes and generalizing findings. Recent literature on retention and attrition finds that longitudinal aging studies struggle to retain race-minority participants, people who are less well-off socioeconomically, and those in poorer health. Such unequal rates of attrition have been reported in the Chicago Health, Aging, and Social Relations Study (CHASRS; Cacioppo & Cacioppo, 2018), the Health and Retirement Study (HRS; Ofstedal & Weir, 2011), The University of Alabama at Birmingham Study of Aging (UAB; Allman et al., 2011), and the Midlife in the United States (MIDUS) study (Radler & Ryff, 2010). The problems associated with samples biased by attrition have led scholars to develop strategies to help mitigate attrition loss (Ofstedal & Weir, 2011) and suggest goals for effective recruitment and retention (Bonk, 2010). However, an issue that has received less attention is how novel retention strategies can be used to reengage people who become lost to a longitudinal panel over time. This article examines rates of attrition across the six latest years (2013–2019) of a 12-year longitudinal panel of older adults in New Jersey and documents how maintaining flexibility in study methodology and employing enhanced retention efforts over time reduced attrition-induced bias. Implications for studying developmental outcomes in aging research are addressed.

### Attrition in Longitudinal Aging Research

Developmental research focuses on understanding change over time in people and identifying factors responsible for the change. However, attrition in longitudinal samples can affect power, limit generalizability, and ultimately affect the way in which change is understood (Hauser, 2005). As Rubin’s missing data framework proposes, missing data can be missing and unrelated to an outcome variable of interest (i.e., missing completely at random), related to variables other than the outcome variable (i.e., missing at random [MAR]), or associated with the dependent variable (i.e., not missing at random [NMAR]; Little & Rubin, 2002). In most cases of developmental science research, missing data are “systematically missing” due to associations of data with other constructs within a model (i.e., MAR or NMAR; Little & Rubin, 2002). Understanding such associations is critical for accurately interpreting developmental change. For example, a sample that loses more men than women over time would present with MAR data, which limits power to examine change or stability for men and may generate findings that are only generalizable to women. Furthermore, the loss of participants who share a distinct set of characteristics that you are trying to study (i.e., NMAR), such as higher levels of pain at baseline and lower perceived subjective well-being, can result in failure to understand how these individuals fare on such constructs as they age. Conclusions may be biased in a positive fashion if more data are lost from people who are initially doing less well.

Evaluating attrition is particularly important when studying older adults because they are a unique subgroup who experience higher levels of impairments such as cognitive, visual, and/or auditory losses, as well as health challenges that can affect their continued participation in research (Lacey et al., 2017; Strotmeyer et al., 2010). Three main reasons for the attrition of older participants in longitudinal studies have been identified: death of the participant, loss of contact with the participant by study staff, and refusal by the participant to continue in the study (Jacomb et al., 2002). Age predicts mortality and lower cognitive function, and age and cognition predict attrition (Cacioppo & Cacioppo, 2018; Stone et al., 2014). Such loss results in a “healthy survivor” effect for remaining people (Murphy et al., 2011). Second, in later life, retirement, widowhood, and major health events can trigger residential mobility, making some groups of people harder to locate (Walters, 2000). Couper and Ofstedal (2006), for example, analyzing data from the HRS and the Panel Study of Income Dynamics (PSID) found that people in households headed by racial minorities and people with lower education had a higher propensity to move. Third, refusal to continue to participate is a challenge in older adult longitudinal panels. In the CHASRS (Cacioppo & Cacioppo, 2018), most withdrawals were due to not wanting to participate, being busy with obligations, and indicating that participation was not worth their time. A small number of studies cited other reasons for sample attrition which were especially relevant to older adults, including health (i.e., too impaired to participate), difficulty scheduling, death of a spouse, hearing impairments, less capacity to understand verbal and written information, and transportation challenges for in-person research (Bonk, 2010; Holt et al., 2015; Lacey et al., 2017).

Beyond these known causes of age-specific attrition patterns, longitudinal aging research studies also report patterns of sample loss similar to those reported by longitudinal studies of younger people (Holt et al., 2015; Iannacchione, 2003). Samples such as the CHASRS (Cacioppo & Cacioppo, 2018), HRS (Ofstedal & Weir, 2011), UAB (Allman et al., 2011), and MIDUS (Radler & Ryff, 2010) report greater loss of people in minority groups, as well as those less well-off economically and clinically.

These findings make the continued study of attrition and retention in large longitudinal samples of older adults important and the identification of strategies that reduce bias critical. Attrition due to death and severe illness cannot be avoided. Some have suggested the need to study such known biased attrition as an outcome in and of itself and to plan analyses knowing that such loss will be evident (Kurland et al., 2009; Murphy et al., 2011; Preston et al., 2013). At the very least, it is important to know who in a panel dies to account for death when analyzing the data and interpreting findings. Moreover, it is critical to understand how people lost to a panel for reasons other than death differ from those not lost in order to reduce attrition and better interpret findings.

### Retention Strategies to Reduce Biased Loss

Concerns about biased loss and retention of people in longitudinal samples are not new to developmental researchers (Bonk, 2010). What is new, are strategies that can help track and retain people or track and reengage participants in a longitudinal aging research panel. Many new strategies for locating participants are direct by-products of advancements in technology. The Internet, for example, has been a key resource for finding individuals for almost 20 years (Passetti et al., 2000). However, web-based search strategies that include using social networking sites such as Classmates, Facebook, Reunion, and MySpace have been used with increasing frequency in the last 10 years (Jones et al., 2012; Masson et al., 2011; Nwadiuko et al., 2011; Perkins et al., 2009; Rhodes & Nasuti, 2011). These social media sites provide a platform for people to post information about their demographics, life events, experiences, and locations on a public web domain. Social media has been effectively used as a tool for tracing, locating, and communicating (via messaging) with participants who are “lost” to follow-up with younger populations (Farabee et al., 2016; Kim et al., 2014; Mychasiuk & Benzies, 2012; Teague et al., 2018). Newer resources also include using people-finder surface web search engines (i.e., search engines that the general public can use and access) such as Google, Yahoo, and Bing or deep-web search engines (i.e., search engines requiring credentials to access) such as Wink, Pipl, PeekYou, Zabasearch, and Intelius. Deep-web search engines like Pipl track changing contact and location information about individuals using a combination of public records, listings, directories, and archives. Such databases can provide email addresses, phone numbers, social media accounts, online accounts, address histories, career and education histories, motor vehicles, professional and leisure associations, and even photos and videos of individuals. Recent empirical evidence documents the utility of accessing information from these sources to track participants in a research study (Cotter et al., 2002; Farabee et al., 2016; Teague et al., 2018). Another strategy includes using telephone and address directories such as Switchboard, Polk City Directories, and Whitepages which provide a public record of phone numbers linked to people. Furthermore, obtaining judicial and death records has become more accessible with online databases (Williams & O’Donnell, 2014).

In conjunction with more sophisticated searching, scholars have benefitted from advances that have enhanced traditional recruitment and retention methods (i.e., regular mailings; Bonk, 2010). The expansion of cell phone carriers with caller ID and texting capabilities has allowed for increased contact (Mitchell et al., 2015), and some work has found that repeated contact by phone (10+ calls) can increase retention (Kleschinsky et al., 2009). Systematic record keeping of detailed contact information with “keep in touch calls” every 3 months and regular mailings (i.e., newsletters and greeting cards) with “Address Service Requested” and expanded staff hours (09:00 a.m. to 09:00 p.m. 7 days a week) have been employed with success (Strawn et al., 2007; The Longitudinal Study of Adult Learning, 2010). Other established longitudinal panels such as HRS, the National Longitudinal Survey of Youth 1979, PSID, the British Household Panel Study, German Socio-Economic Panel, and Household, Income and Labor Dynamics in Australia Survey have relied on incentive payments, field-based strategies, and survey design features to retain samples (Schoeni et al., 2013). For African American and Hispanic subgroups, HRS has utilized an oversampling approach that has helped to maintain an adequate minority sample despite attrition (Ofstedal & Weir, 2011). Studies of Mexican Americans have used an individualized approach for collecting information based on life events that each person has experienced to keep track of their whereabouts with some success (Ortiz & Ballon, 2007). The Leiden 85-plus study used home visits to collect data from sample members who initially refused participation (Bootsma-van der Wiel et al., 2002), and the Cardiovascular Health Study utilized home visits in addition to telephone and proxy visits to improve retention (Strotmeyer, 2010).

### Present Study

This article has three goals, each of which expands knowledge about attrition in longitudinal aging studies. Goals include (a) examining omnibus group differences between people lost to follow-up in a state-wide panel of older adults and people who continue to participate (completers) in terms of demographic characteristics (i.e., race, income, and age) and indicators of successful aging (i.e., subjective successful aging, chronic illnesses, functional ability, and pain); (b) determining how people lost to follow-up for different reasons (i.e., become impaired, lack interest, die, and lack follow-up information) compare with completers on demographic indicators and successful aging attributes; and (c) examining the impact of expanded innovative retention strategies to mitigate biased loss over time by comparing different types of completers (i.e., full completers, those lost to follow-up, and then reengaged) on demographic indicators and successful aging attributes. These analyses add to the literature on longitudinal aging research regarding retention by documenting attrition patterns in a large-scale longitudinal panel of older adults and identifying the differential patterns of participation following expanded retention efforts—including full completers and partial completers (those lost at prior waves and later reengaged). Implications for interpretation of results in successful aging research are discussed.

## Research Design and Methods

### Sample Recruitment and Retention Strategies

These analyses use the ORANJ BOWL (Ongoing Research on Aging in New Jersey—Bettering Opportunities for Wellness in Life) longitudinal research panel. As this panel developed, resources and staffing ebbed and flowed, providing a natural experiment from which much can be learned about attrition and the impact of expanded retention strategies.

ORANJ BOWL began with a random-digit-dial sample of 5,688 community-dwelling older adults in New Jersey who were interviewed between November 2006 and April 2008 (Wave 1). Eligibility included being between the ages of 50 and 74, living in New Jersey, and having the ability to participate in a 1-h, English-language telephone interview. Details about sample recruitment can be found in the work of Pruchno et al. (2010). The panel was representative of older people living in New Jersey in 2006, except for a slightly higher rate of women and people with more years of education. Because we lacked the resources needed to translate the interview into Spanish, ORANJ BOWL underrepresents Hispanics.

From the onset, a distinct feature of the ORANJ BOWL panel retention efforts was its branding. The panel’s name and logo (a football with six stick-figures of varying shades from white to brown and black) depicted participants and researchers as a team focused on learning about successful aging. Upon completion of the baseline interview, ORANJ BOWL participants were sent a membership kit that included a welcome letter signed by hand, a plastic membership card, a gift of a postage stamp sheet, and a trifold informational pamphlet—all with the service-marked ORANJ BOWL logo displayed. The informational pamphlet described the research goals and provided details using the terms “team” and “players” to describe the panelists. All subsequent correspondence used this branding approach, displayed the ORANJ BOWL logo, and called upon participants to continue to participate as a “team” member (i.e., newsletters, holiday cards, and birthday cards); the study website and toll-free numbers used the ORANJ BOWL acronym. Small gifts of appreciation were sent upon survey completion which emphasized team membership in lieu of monetary compensation: postage stamps (baseline), deck of playing cards (Wave 2), grocery tote bag (Wave 4), umbrella (Wave 5), jar opener grippers (holiday gift between Waves 5 and 6), small notepad with a pen (Wave 6), and luggage grippers (post-Wave 6).

Between 2007 and 2008, the first 2,674 participants recruited to the panel were recontacted 1 year after their baseline interview and asked to complete a personality inventory (Wave 2). In 2011, a questionnaire was mailed to all ORANJ BOWL respondents (Wave 3). This wave was funded by a small grant from the University of Medicine and Dentistry of New Jersey (UMDNJ) Foundation. Funding cuts (at Wave 2) and limited resources (at Wave 3) did not allow for retention activities at these waves. As such, the retention analyses that follow do not include these waves.

In 2013, funding from the Rockefeller Foundation allowed for the mailing of a questionnaire (Wave 4) focused on the effects of Hurricane Sandy to all ORANJ BOWL respondents known to be alive at Wave 3. Then, funding from Assistant Secretary for Preparedness and Response enabled us to hire a small staff to call nonresponders and to complete the interview by telephone. To encourage participation, nonresponders were mailed a $1 incentive with a hard copy of the questionnaire, a strategy invoked by others with success (Beebe et al., 2005; Dillman et al., 2009; Singer, 2002). Additional retention strategies included mailing birthday postcards and holiday cards, reaching out to secondary contacts (provided by participants at baseline), and tracing hard-to-find participants using free online databases (i.e., Google).

Wave 5 data were collected approximately 18 months after Wave 4 (2015–2017). Funding from the National Institute on Aging (NIA), for a study focused on the effects of Hurricane Sandy on the functional limitations of older people (R01 AG046463), enabled us to hire more staff and bolster retention efforts. Consistent with other longitudinal studies, we used retention strategies that included sending birthday cards, holiday cards, and newsletters throughout the year. All of these mailings included a statement for “Address Service Requested” on return addresses, helping staff keep addresses current. Our team developed a standardized 8-week procedure for locating participants. Advance letters were bunched and mailed to the addresses on record informing participants that an interviewer would call them within a week. Interviewers called all available phone numbers multiple times per day. If interviewers could not reach a participant, they would try calling a secondary contact (i.e., family member or friend), identified by each respondent at baseline and then at each subsequent interview as someone who would always know the participant’s whereabouts. We gave panelists the choice of completing the interview by telephone with an interviewer or independently online using Qualtrics. People who could not complete the questionnaire by either of these modes (usually because they were hard of hearing or did not have access to a computer) were offered the opportunity to complete the questionnaire using a hard copy that was sent and returned by mail. Other studies had shown that a flexible mode of administering a questionnaire is an effective means of improving response rate (Beebe et al., 2005; Dillman et al., 2009; Griffin & Obenski, 2002).

Wave 6 data, also funded by NIA, were collected approximately 18 months after the completion of Wave 5 (2017–2019). We continued using the protocols we had established in Wave 5 which were used by well-established longitudinal studies for locating participants (Hauser, 2005; Kleschinsky et al., 2009; Passetti et al., 2000; Stone et al., 2014; Williams & O’Donnell, 2014). However, as staff began to work with Wave 5 data, we found that over time, the ORANJ BOWL panel was experiencing the same biased loss identified in other panels. People most likely to be lost from the panel were members of race-minority groups, those with lower income, and people with more health issues at baseline, characteristics likely to affect the generalizability of findings (Heid et al., 2018).

We addressed this issue by launching a pilot study. We randomly selected 125 people who had not responded to retention efforts at Wave 5 (many of whom had not participated since baseline) and developed new tactics to reengage these people at Wave 6. We began by extending interviewing hours, adding staff who could interview in the evenings and on weekends. Second, we gave participants the opportunity to complete the interview in multiple sittings. Third, we began using new tools for locating participants, including web-based surface search strategies (Google name, state, and obituary; That’s Them, NJ property/parcel tax records, Standard White Pages, 411, Advance Background Checks, Spydialer, Hometry, and Ecolisting.com for Comcast users), commercial deep-web search engines (Been Verified and Instant Checkmate), and social media (Facebook, MySpace, Classmates, and Twitter). Fourth, we developed a unique team-based approach for locating participants in which research assistants worked cases together. Each team comprises three research assistants who worked varying shifts/days: morning, mid-morning to afternoon, and evening/weekend shifts. One research assistant would call a participant during their working hours for 2 weeks. If there was no response, that research assistant would pass the case to the second teammate. If the team was not able to contact the participant, the third teammate would send a letter by snail and/or email to inform them that the study team had been calling with no success. Then the third research assistant would make two to three more calls. If no contact was made, the case was returned back to the original research assistant to contact the secondary contact on file. After Week 8, the case was passed to a research assistant known as the “tracer” to complete a deep-web search for additional contact information. Participants with nonworking telephone numbers were directly assigned to a “tracer.” If a tracer found additional contact information, the call process was then restarted, and the established protocol was followed until a final status was determined.

The pilot study showed that this process proved effective and feasible. We were able to locate 79% (*N* = 99) of the sample of 125 that had been considered lost to follow-up with the expanded retention strategies. More specifically, 30% (*N* = 38) completed the interview, 8% (*N* = 10) were confirmed as deceased, 12% (*N* = 15) were determined to be too ill/impaired, 27% (*N* = 34) withdrew due to lack of interest, and 2% (*N* = 2) moved out of the country. After these expanded efforts, only 21% (*N* = 26) of the pilot subsample remained unable to locate. As a result of this success, we implemented the full expanded retention effort to all remaining cases at Wave 6.

**Figure 1.**
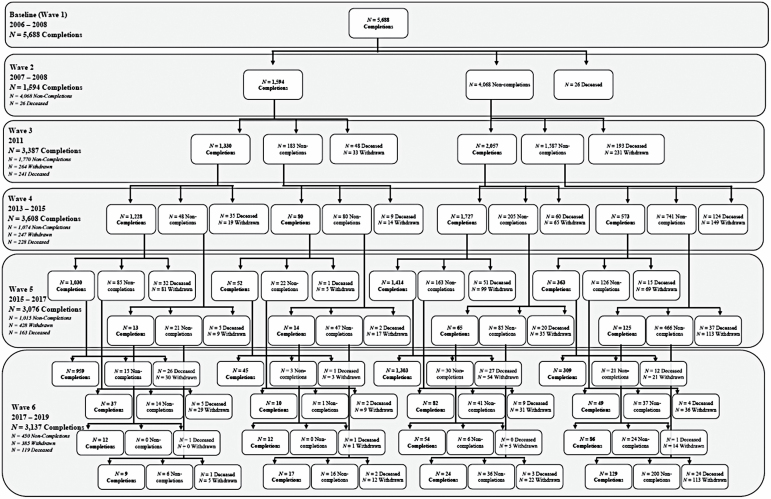
ORANJ BOWL (Ongoing Research on Aging in New Jersey—Bettering Opportunities for Wellness in Life) sample completion flow chart across six waves of data collection.

Sample sizes and completion flow are shown in Figure 1. Demographic information was collected at baseline. At baseline and each subsequent wave, panelists answered closed-ended questions about successful aging. Institutional Review Board approval was obtained from UMDNJ and Rowan University for all data collection procedures.

### Measures

#### Demographic characteristics

Participants reported their age, education, race, gender, marital status, work status, and income at baseline (2006–2008). Education was coded 1 (*not a high school graduate*) to 9 (*masters, doctorate, or professional degree*). Race was coded 0 (*not Black/African American*) or 1 (*Black/African American*). Gender was coded 0 (*man*) or 1 (*woman*). Income was coded 1 (*less than $30,000*) to 6 (*more than $150,000*). Marital status was coded as 1 (*married*) or 0 (*not married*). Work status was coded as 0 (*fully retired*), 1 (*working full-time*), 2 (*working part-time*), 3 (*a homemaker*), 4 (*in school*), 5 (*disabled*), 6 (*unemployed looking for work*), and 7 (*unemployed not looking for work*). For work status, we compared all others to those working full-time.

#### Successful aging

In prior work, we established the validity of a two-factor definition of successful aging that includes indicators of objective and subjective success (Pruchno et al., 2010). *Subjective successful aging* was assessed at each wave with questions that had respondents evaluate themselves using a scale from 0 to 10. Respondents reported what number best (a) describes how successfully they have aged, (b) describes how well they are aging, and (c) represents how they would rate their life these days. A total score was created by summing responses. Objective successful aging was measured with three indicators. First, was a count of *chronic illnesses* (arthritis, hypertension, a heart condition, cancer, diabetes, osteoporosis, stroke, and lung conditions) that had ever been diagnosed by a physician or health professional. We focused on these eight conditions because they are chronic and are typically associated with age (range 0–8). Second, *functional ability* was measured using nine items that asked participants to rate their upper and lower body capabilities on a scale from 1 (*you can’t do it at all*) to 5 (*not at all difficult*). Items were summed to create a total score of functional ability (α = 0.92 at baseline; range 9–45). Finally, *pain* was measured with the following three questions: “How often are you troubled with pain?”; “How bad is the pain most of the time?” and “How often does the pain make it difficult for you to do your usual activities such as household chores or work?” Each question used a 4-point Likert response scale from 0 (*low*) to 3 (*high*) pain. A total score of pain was created by summing responses (α = 0.88 at baseline; range 0–9).

### Analysis Plan

The analyses that follow are focused on Waves 4, 5, and 6, as we had sufficient resources at these waves to support retention activities. As a preliminary step, sample disposition was documented at each wave. Consistent with past research on attrition (Cacioppo & Cacioppo, 2018; Jacomb et al., 2002), we classified four reasons for attrition: *Death*, *Impairment*, *Not interested*, and *Noncompleters*. *Deaths* were reported by next of kin and/or identified in a newspaper obituary or National Death Index search. *Impaired* participants included people who (a) self-reported a diagnosis of Alzheimer’s disease or other cognitive impairment, (b) failed a cognitive screen used at Waves 5 and 6 (Telephone Interview of Cognitive Status - Modified version score of <28; Brandt et al., 1988; Breitner et al., 1990; Welsh et al., 1993), (c) were blind or had major vision problems, (d) said they were too ill to participate in the study (i.e., terminal illness), and (e) had moved to a nursing home. *Not interested* participants included people who said they (a) were no longer interested in participating, (b) were too busy, (c) moved out of the country, (d) could not remember the study, or (e) wanted to withdraw with no reason provided. The *Noncompleter* category included people who (a) were alive but not responding to study staff (i.e., avoiders), (b) staff were unable to locate at a given wave (i.e., people we could not verify as alive or for whom we could not verify contact information at that wave), and (c) opted to skip the interview for no reason or for reasons such as not interested at the time, too busy, or too ill for the given interview.

In addition to these four reasons cited by others, we identified people lost to the panel for a fifth reason—*Untraceable*. *Untraceable* participants included those who did not provide us with enough information in the baseline interview to identify and contact for subsequent follow-up (i.e., full name, address, and/or date of birth).

We computed completion rates using algorithms used by others (Radler & Ryff, 2010), including (a) a raw completion rate (% of prior active sample who completed), (b) an adjusted completion rate that removed individuals who could not participate from the denominator (% of prior active sample minus those determined deceased or lost to illness/impairment who completed), and (c) a final “located rate” for each wave (% of active sample whose disposition was determined, regardless of completion status). We adjusted completion rates for illness/impairment in addition to death given that participants withdrawn from the sample for these reasons were no longer considered eligible to participate in subsequent waves.

To examine the overall effects of attrition on key demographic characteristics and measures of successful aging, we ran unadjusted comparisons between active participants (those completing Wave 6) and all people lost to follow-up, regardless of the reason for loss. The sample was split into two groups—active participants as of Wave 6 (i.e., completers) and inactive as of Wave 6 (i.e., lost to follow-up). Next, to examine how completers differed from noncompleters as a function of the reason for noncompletion, we ran unadjusted comparisons for demographic characteristics and successful aging attributes using information about the reason for loss (Deceased, Impaired, Not interested, Untraceable, and Noncompleters). Finally, we compared demographic characteristics and successful aging attributes for different types of completers based on participation status at Waves 4, 5, and 6. We ran unadjusted comparisons, splitting people into those who had (a) completed Wave 4, Wave 5, and Wave 6 interviews (full completers) and (b) not completed the Wave 4 and/or Wave 5 interview but did complete the Wave 6 interview (people we successfully reengaged in the panel). We also ran descriptive statistics specifically on participants who did not complete Wave 5 but returned for Wave 6 to determine demographic distributions within this subsample and the effectiveness of our expanded retention efforts.

We used generalized linear models (PROC GLM) to compare continuous constructs and chi-square tests of difference for categorical constructs. Of note, education and income were interval variables treated as continuous constructs due to normal distributions and coverage across the range of responses within the sample.

## Results

Figure 1 details completion status across waves. *Impaired* and *Not interested* participants are classified as withdrawn. Table 1 presents raw completion rates, adjusted completion rates, and location rates at each wave of analysis. ORANJ BOWL retained between 71% (Wave 5) and 82% (Wave 6) of the sample. With added resources and focused retention efforts, we were able to increase the location rate from 81% at Wave 4 to 90% at Wave 6.

**Table 1.** Completion Rates by Wave

Wave	Raw completion rate^a^	Adjusted completion rate^b^	Location rate^c^
Wave 4	70% (3,608/5,157)	74% (3,608/4,876)	81% (4,169/5,157)
Wave 5	66% (3,076/4,682)	71% (3,076/4,340)	80% (3,742/4,682)
Wave 6	77% (3,137/4,091)	82% (3,137/3,844)	90% (3,687/4,091)

^a^% of prior active sample who completed.

^b^% of prior active sample minus those determined lost to death and illness who completed.

^c^% of active sample whose disposition was determined, regardless of completion status.

In regard to our first goal, we found that at baseline, people who completed the Wave 6 interview differed from those lost to follow-up. Specifically, as seen in Table 2, people who completed the Wave 6 interview were significantly younger, less likely to be African American, more likely to be married, reported higher levels of income and education, were more likely to be working full-time, had less pain and fewer chronic illnesses, and reported better functional ability and subjective successful aging at baseline than those lost to follow-up. Additional analyses reveal that differences between completers and those lost to follow-up at each wave (4, 5, and 6) are similar to those at Wave 6 (analyses available from authors upon request).

**Table 2.** Comparison of Demographics and Successful Aging Outcomes for Wave 6 Completers and All Other Participants Lost to Follow-Up as of Wave 6

Participant characteristic	Completers at T6 *N* = 3,137	Lost to follow-up as of T6 *N* = 2,551	Group differences
Age, *M* (*SD*)	59.70 (6.68)	62.15 (7.37)	*F*(1, 5,686) = 172.49, *p* < .001
Gender, % (*N*) male	36.4% (1,141)	36.3% (926)	NS^a^
Race, % (*N*) African American	8.2% (250)	16.1% (396)	χ ^2^ (4, *N* = 5,522) = 105.29, *p* < .001
Marital status, % (*N*) married	62.3% (1,954)	49.7% (1,267)	χ ^2^ (1, *N* = 5,681) = 90.91, *p* < .001
Income, *M* (*SD*)^b^	4.28 (1.34)	3.50 (1.52)	*F*(1, 5,020) = 372.53, *p* < .001
Education, *M* (*SD*)^c^	4.49 (2.11)	3.50 (2.03)	*F(*1, 5,673) = 318.17, *p* < .001
Work status, % (*N*) employed full-time	49.6% (1,557)	33.7% (858)	χ ^2^ (8, *N* = 5,681) = 258.48, *p* < .001
Pain, *M* (*SD*)^d^	2.33 (2.42)	2.94 (2.78)	*F*(1, 5,682) = 78.54, *p* < .001
Chronic illnesses, *M* (*SD*)^e^	1.57 (1.28)	1.99 (1.50)	*F*(1, 5,686) = 126.80, *p* < .001
Functional ability, *M* (*SD*)^f^	41.26 (5.45)	38.64 (7.45)	*F*(1, 5,686) = 232.76, *p* < .001
Subjective successful aging, *M* (*SD*)^g^	23.88 (4.06)	22.86 (4.95)	*F*(1, 5,681) = 73.00, *p* < .001

^a^NS = not significant.

^b^Income was coded 1 (*<$30,000*) to 6 (*>$150,000*).

^c^Education was coded 1 (*not a high school graduate*) to 9 (*masters, doctorate, or professional degree*).

^d^Pain was assessed with a three-item scale, range 0–9.

^e^Chronic illnesses include a count of eight illnesses, range 0–8.

^f^Functional ability was assessed with a nine-item scale, range 9–45.

^g^Subjective successful aging was assessed with a three-item scale, range 0–30.

Findings addressing our second goal (to determine how individuals lost to follow-up for different reasons differ from completers on demographic indicators and successful aging attributes) are reported in Table 3. We found that baseline characteristics of panel members who completed Wave 6 varied as a function of the reason people were lost to follow-up. Completers were significantly older, less likely to be African American, more likely to be married, had higher levels of income and education, reported less pain, and greater functional ability at baseline than participants who were classified as *noncompleters* (i.e., unable to locate, not responding/avoiding, skips). Completers were significantly younger, less likely to be African American, more likely to be married, had greater income and education, more likely to work full-time, had fewer chronic conditions, less pain, and higher levels of subjective successful aging and functional ability at baseline than those lost to *impairment* or *death*. Completers had higher levels of income and education and greater functional ability compared to those who withdrew because they were *no longer interested*. And finally, completers were significantly younger than *untraceable* participants.

**Table 3.** Demographics and Successful Aging Outcomes by Completion Status as of Wave 6 for Completers Versus Individuals Lost to Follow-Up for Different Reasons

	(0)	(1)	(2)	(3)	(4)	(5)	
Participant characteristic	Completers, *N* = 3,137	Noncompleter, *N* = 450	Impaired, *N* = 381	Not interested, *N* = 832	Deceased, *N* =777	Untraceable, *N* = 111	Group differences
Age, *M* (*SD*)	59.70 (6.68)	57.99 (6.34)	65.80 (6.60)	60.33 (7.04)	64.79 (6.79)	61.92 (7.06)	*F*(5, 5,682) = 132.33^***^
							Group 0 < 2^***^, 4^***^, 5^**^
							Group 0 > 1^***^
Gender, % (*N*) male	36.4% (1,141)	36.4% (164)	34.6% (132)	32.8% (273)	41.2% (320)	33.3% (37)	χ ^2^ (5, *N* = 5,688) = 13.27*
Race, % (*N*) African American	8.2% (250)	22.2% (96)	22.3% (80)	9.8% (79)	17.7% (134)	6.7% (7)	χ ^2^ (20, *N* = 5,688) = 236.07^***^
Marital status, % (*N*) married	62.3% (1,954)	51.2% (230)	46.5% (177)	59.6% (495)	39.4% (306)	53.6% (59)	χ ^2^ (5, *N* = 5,688) = 159.93^***^
Income, *M* (*SD*)^a^	4.28 (1.34)	3.68 (1.44)	2.99 (1.45)	3.97 (1.44)	3.08 (1.51)	4.13 (1.44)	*F*(5, 5,016) = 119.60^***^
							Group 0 > 1^***^, 2^***^, 3^***^, 4^***^
Education, *M* (*SD*)^b^	4.49 (2.11)	3.57 (2.04)	3.09 (1.96)	3.77 (2.06)	3.26 (1.92)	4.29 (2.19)	*F*(5, 5,669) = 75.39^***^
							Group 0 > 1^***^, 2^***^, 3^***^, 4^***^
Work status, % (*N*) employed full-time	49.6% (1,557)	50.7% (228)	19.7% (75)	42.5% (353)	19.8% (154)	44.0% (48)	χ ^2^ (40, *N* = 5,688) = 601.74^***^
Pain, *M* (*SD*)^c^	2.33 (2.42)	2.85 (2.66)	3.13 (2.80)	2.50 (2.52)	3.43 (3.02)	2.55 (2.64)	*F*(5, 5,676) = 27.41^***^
							Group 0 < 1^**^, 2^***^, 4^***^
Chronic illnesses, *M* (*SD*)^d^	1.57 (1.28)	1.51 (1.35)	2.30 (1.46)	1.60 (1.34)	2.58 (1.55)	1.57 (1.37)	*F*(5, 5,682) = 87.25^***^
							Group 0 < 2^***^, 4^***^
Functional ability, *M* (*SD*)^e^	41.26 (5.45)	40.20 (6.39)	37.98 (7.50)	40.36 (6.27)	35.99 (8.45)	40.26 (6.15)	*F*(5, 5,682) = 96.25^***^
							Group 0 > 1*, 2^***^, 3^**^, 4^***^
Subjective successful aging, *M* (*SD*)^f^	23.88 (4.06)	23.30 (4.50)	22.74 (4.86)	23.63 (4.25)	21.72 (5.65)	23.68 (5.14)	*F*(5, 5,677) = 31.69^***^
							Group 0 > 2^***^, 4^***^

^a^Income was coded 1 (*<$30,000*) to 6 (*>$150,000*).

^b^Education was coded 1 (*not a high school graduate*) to 9 (*masters, doctorate, or professional degree*).

^c^Pain was assessed with a three-item scale, range 0–9.

^d^Chronic illnesses include a count of eight illnesses, range 0–8.

^e^Functional ability was assessed with a nine-item scale, range 9–45.

^f^Subjective successful aging was assessed with a three-item scale, range 0–30.

*p < .05, **p < .01, ***p < .001.

Finally, comparing full completers (at Waves 4, 5, and 6) to completers who were lost to follow-up (at Waves 4 and/or 5) but then reengaged (at Wave 6), we found that individuals completing all three waves were significantly older, less likely to be African American, more likely to be married, less likely to be working full-time, had higher income and education, reported less pain and higher levels of functional ability and subjective successful aging at baseline than individuals not completing Wave 4 and/or Wave 5 but completing Wave 6 (Table 4). People who were lost and reengaged were significantly different from those who remained within our sample over time. Looking more specifically at those that did not complete Wave 5 but did complete Wave 6, we found that 36% of this sample was a high school graduate or less, 23% had an income of $30,000 or less, and 19% were African American, affirming that enhanced retention efforts were successful in bringing back a greater proportion of individuals with less education and/or income and African American participants than traditional retention efforts.

**Table 4.** Comparison of Demographics and Successful Aging Outcomes for Full Completers (at Waves 4, 5, and 6) and Those Lost to Follow-Up (at Wave 4 and/or 5) and Reengaged (at Wave 6)

Participant characteristic	Full completers (at T4, T5, and T6), *N* = 2,616	Lost and reengaged (lost at T4 and/or T5, completed T6), *N* =521	Group differences
Age, *M* (*SD*)	59.96 (6.69)	58.36 (6.45)	*F*(1, 3,135) = 25.03, *p* < .001
Gender, % (*N*) male	36.3% (949)	36.9% (192)	NS^a^
Race, % (*N*) African American	6.5% (167)	16.6% (83)	χ ^2^ (4, *N* = 3,064) = 70.15, *p* < .001
Marital status, % (*N*) married	63.7% (1,665)	55.6% (289)	χ ^2^ (1, *N* = 3,134) = 12.18, *p* = .001
Income, *M* (*SD*)^a^	4.34 (1.30)	3.96 (1.48)	*F*(1, 2,807) = 31.59, *p* < .001
Education, *M* (*SD*)^b^	4.60 (2.11)	3.95 (2.01)	*F*(1, 3,132) = 41.23, *p* < .001
Work status, % (*N*) employed full-time	48.8% (1,277)	53.7% (280)	χ ^2^ (8, *N* = 3,136) = 36.783, *p* < .001
Pain, *M* (*SD*)^c^	2.23 (2.35)	2.84 (2.67)	*F*(1, 3,133) = 28.09, *p* < .001
Chronic illnesses, *M* (*SD*)^d^	1.57 (1.26)	1.60 (1.37)	NS
Functional ability, *M* (*SD*)^e^	41.48 (5.11)	40.13 (6.81)	*F*(1, 3,135) = 26.84, *p* < .001
Subjective successful aging, *M* (*SD*)^f^	24.05 (3.92)	23.03 (4.58)	*F*(1, 3,131) = 27.50, *p* < .001

^a^NS = not significant.

^a^Income was coded 1 (*<$30,000*) to 6 (*>$80,000*).

^b^Education was coded 1 (*not a high school graduate*) to 9 (*masters, doctorate, or professional degree*).

^c^Pain was assessed with a three-item scale, range 0–9.

^d^Chronic illnesses include a count of eight illnesses, range 0–8.

^e^Functional ability was assessed with a nine-item scale, range 9–45.

^f^Subjective successful aging was assessed with a three-item scale, range 0–30.

## Discussion and Implications

Biased attrition in longitudinal aging research is a concern, particularly for scholars seeking to understand developmental outcomes. In this article, we examined rates and reasons for attrition within the six-wave, ORANJ BOWL research panel, a state-wide sample of older adults in New Jersey. We found that participants completing the final wave of ORANJ BOWL data collection (i.e., Wave 6 completers) were distinct from those lost to follow-up at Wave 6 regarding age, race, marital status, income, education, and full-time work status. Completers also reported better objective and subjective successful aging at baseline compared to individuals lost to follow-up. Second, we were able to delineate how individuals who were lost to follow-up for different reasons differed from completers, based on the reason for loss. Third, we showed that implementing expanded retention efforts decreased sample bias. Participants who were lost to the study and reengaged were younger, more likely to be African American, not married, had lower income and education, and were more likely to be working full-time. These people reported lower baseline subjective successful aging and functional ability and higher pain. These findings collectively carry implications for interpretation of research and designs of future longitudinal research in aging.

The first main finding is consistent with others’ work with longitudinal panels of older adults (Cacioppo & Cacioppo, 2018; Ofstedal & Weir, 2011; Radler & Ryff, 2010; Strotmeyer et al., 2010). Completers and those lost to follow-up are different. Over time, individuals who are older, African American, not married, and/or have lower income or education or not working full-time were more likely to be lost to follow-up. Findings from this work demonstrate that participants lost were also significantly different from completers depending upon their reason for loss. They differed on demographic characteristics but also on reports of successful aging components at baseline. Those lost reported more pain, more chronic illnesses, lower scores on subjective successful aging, and lower levels of functional ability at baseline. The biased loss was in part due to the loss of participants from uncontrollable attrition loss (impairment/death). Those who died or were too impaired were as expected, older, more likely to be African American, not married, had lower income and/or education, and were less likely to be working full-time, but also had more chronic conditions, more pain, and lower subjective successful aging and functional ability at baseline.

However, we also found that participants who completed were distinct from groups of participants who attrite due to reasons that may, in practice, be more controllable. People who were untraceable were older. Those not responding/unable to locate/skipped were also older, more likely to be African American, less likely to be married, had lower income and/or lower education, reported more pain, and less functional ability at baseline. These findings are consistent with prior work (Couper & Ofstedal, 2006; Walters, 2000) and provide insight into the types of people who may require more intensive or altered tracking. Initial recruitment efforts may need to expand modes of contact (i.e., add an in-person/home-visit component) or data tracking on such subsamples to reduce a later loss (Bonk, 2010; Mody et al., 2008; Strotmeyer et al., 2010). Furthermore, those lost due to lack of interest were also individuals with lower income/education and less functional ability at baseline. The survey structure may need to be modified for this group to increase appeal.

Findings from this study provide unique information about successful aging as completers and those lost to follow-up differed on attributes of successful aging. As scholars seek to understand the developmental course of successful aging (Pruchno & Wilson-Genderson, 2015), we must take into account who we are truly studying over time (i.e., “healthy survivors,” those who could still be located, those who still opted to participate; Preston et al., 2013). The conclusions we draw are specific to these individuals. Given that loss is greatest in those with lower reports of subjective success, higher pain, and lower functional ability, samples may be losing those who are “unsuccessful” from the start (i.e., data are NMAR). Our samples are limited in range, and exercises that seek to distinguish groups of individuals who are aging successfully over time may only be considering those who are to some degree “successful.” Perhaps as suggested by other scholars (Kurland et al., 2009; Murphy et al., 2011; Preston et al., 2013), we must focus our energy on studying “uncontrollable” attrition reasons (i.e., impairment and death) and design our analyses to account for specific types of loss to truly understand why some individuals are able to survive and achieve “success” compared to others who are lost.

Furthermore, a novel finding from this work is that implementing new creative retention strategies as a longitudinal panel ages can reduce biased loss. Utilization of not one strategy but a combination of newer efforts (i.e., social media, deep-web searches, and a team-based approach) allowed us to reengage a greater number of African American participants, those who were younger and more likely to be working full-time, as well as those who had lower income, education, and ratings of successful aging. As proposed by others, reviewing study methodology and modifying approaches to align with the needs and capabilities of panel participants over time improved retention (Mody et al., 2008). Like other studies, we found that there is no single “magic bullet” that will keep people participating in panels, rather persistence and utilization of a combination of strategies was key in reengaging/retaining the sample (Ortiz & Ballon, 2007; Strawn et al., 2007; Williams & O’Donnell, 2014). In a systematic meta-analysis of 143 longitudinal cohort studies, Teague et al. (2018) identified 95 retention strategies broadly classified as either barrier-reduction, community-building, follow-up/reminder, or tracing strategies. Yet our results demonstrate that in addition to successfully using tracing strategies, specifically employing strategies aimed to reduce participation burden (e.g., flexibility from a research team in scheduling, skipping waves, and splitting data collection over multiple sessions) improved retention. This finding underscores the critical need for allotting financial resources to retention training and retention efforts in longitudinal research that allow for use of multiple tracking databases and increased interviewer time on cases (Bonk, 2010).

This work is not without limitation. Because retention strategies were combined, we were unable to document the direct impact of each specific strategy employed (i.e., Facebook tracking or deep-web searching). As a result, the direct impact of each strategy on reduced attrition is unknown. Second, although there was a representative sample of African American individuals in New Jersey at baseline, the sample did not include a representative sample of Hispanic and other racial minorities. Results from this work, therefore, do not apply to other racial groups. Different retention strategies may prove more useful for retaining or reengaging different minority groups in longitudinal research.

Overall, this study advances our understanding of the utility of retention strategies for longitudinal aging studies seeking to understand developmental outcomes by demonstrating how changes in retention strategies over time can reduce sample bias. We also demonstrate that successful aging indicators are differentially reported for those who continue to participate over time and those who do not. Such a finding carries implications for how we interpret longitudinal findings and design studies of successful aging in the future. Future work must continue to employ efforts that reduce biased loss and examine factors that are associated with “uncontrollable” loss (impairment/death) to understand successful aging.
